# Association between vitamin D level and hematuria from a dipstick test in a large scale population based study: Korean National Health and nutrition examination survey

**DOI:** 10.1186/s12882-019-1369-z

**Published:** 2019-05-24

**Authors:** Hyunjin Ryu, Hyunjeong Cho, Yun Kyu Oh, Kwon Wook Joo, Yon Su Kim, Curie Ahn, Seung Seok Han

**Affiliations:** 1 Department of Internal Medicine, Seoul National University Hospital, 101, Daehak-ro, Jongno-gu, Seoul, 03080 Republic of Korea; 2 Department of Internal Medicine, Chungbuk National University Hospital, 776, 1sunhwan-ro, Seowon-gu, Cheongju-si, Chungcheongbuk-do 28644 Republic of Korea; 3 Department of Internal Medicine, Seoul National University Boramae Medical Center, 20, Boramae-ro 5-gil, Dongjak-gu, Seoul, 07061 Republic of Korea; 4 Department of Internal Medicine, Seoul National University College of Medicine, 103 Daehak-ro, Jongno-gu, Seoul, 03080 Republic of Korea

**Keywords:** Vitamin D deficiency, Hematuria, Sex, Menopause

## Abstract

**Background:**

Vitamin D deficiency is an important health concern because it is related to several comorbidities and mortality. However, its relationship with the risk of hematuria remains undetermined in the general population. In this study, we analyzed the association between vitamin D deficiency and hematuria.

**Methods:**

We conducted cross-sectional analysis using data of participants from the Korean National Health and Nutrition Examination Survey (KNHANES) 2010–2014. A total of 20,240 participants, aged ≥18 years old, were analyzed. Serum 25-hydroxyvitamin D (25(OH)D) levels were measured in a central laboratory and hematuria was defined as ≥1+ on a dipstick test. Multivariate logistic regression was conducted to calculate the odds ratio (OR) of hematuria risk according to serum 25(OH)D quartiles, after adjusting several covariates.

**Results:**

A total 3144 (15.5%) participants had hematuria. The mean 25(OH)D level was 17.4 ± 6.2 ng/mL (median, 16.6 ng/mL (interquartile range, 13.1–20.8 ng/mL)). The 3rd and 4th quartiles had a higher risk of hematuria than the 1st quartile, with adjusted ORs 1.26 (1.114–1.415) and 1.40 (1.240–1.572) in the 3rd and 4th quartiles, respectively. However, this relationship was only significant in women, not in men. When stratified analyses were conducted according to menopausal status, there was a significant increase of hematuria risk according to quartiles in postmenopausal but not in premenopausal women.

**Conclusion:**

We found that vitamin D deficiency is correlated with hematuria in women, particularly after menopause. Further interventional studies are warranted to address whether correcting vitamin D deficiency can lower the risk of hematuria.

## Background

Vitamin D has receptors that are expressed in many nucleated cells and controls the expression of various human genes [1]. Vitamin D deficiency aggravates bone diseases, leading to osteoporosis, and increases the risk of falls and fractures [2, 3]. In addition to its relationship with skeletal health, the association of vitamin D deficiency and various other diseases such as hypertension [4], cardiovascular disease [5–7], cancer [8–11], infectious disease, and metabolic disease [12] have also received attention. Vitamin D deficiency is a global health problem related to poor nutrition [2], and the prevalence of vitamin D deficiency is relatively high worldwide. According to data from the National Health and Nutrition Examination Survey of the United States, 10–40% of the population is deficient in vitamin D [13]. The prevalence of vitamin D deficiency is even higher among Asians than in the United States [2]. According to a Korean report, 47.3% of males and 64.5% of females are deficient in vitamin D [14]. Correcting vitamin D deficiency is essential to preventing several related diseases and improving global human health.

Hematuria is the presence of red blood cells in the urine. The prevalence of hematuria ranges from 0.2 to 16.1% in the general population [15, 16]. In one study, 6.2% of Korean participants who underwent health screening had asymptomatic hematuria [17]. Hematuria is frequently the result of nonglomerular causes, such as an infection or stone in the urinary tract. Additionally, hematuria can be a manifestation of glomerular kidney disease or polycystic kidney disease and is known to be a risk factor of progressive kidney dysfunction and end-stage renal disease [18]. Various urinary tract neoplasms originating in the bladder, prostate, ureter, and kidney may manifest as microscopic and gross hematuria [19]. Therefore, hematuria is an important sign of disease and its cause should be evaluated to prevent further disease progression.

Despite the clinical importance of vitamin D deficiency and hematuria, no studies have been conducted to investigate their correlation in the general population. Correlation between proteinuria and vitamin D deficiency has been evaluated in various studies [20, 21]. However, the association between vitamin D status and the hematuria, another important parameter of kidney disease other than proteinuria, has not been evaluated yet. Furthermore, there are accumulating evidence that vitamin D deficiency contributes to pathologic conditions that can be presented as hematuria such as urinary stone [22], infection [23] and malignancy [24]. The present study is the first to examine this correlation using data of a nationwide population-based survey, stratified by sex and menopause status as these parameters are known to be important in analyzing the effects of vitamin D deficiency [25, 26].

## Methods

### Study population

This was a nationwide population-based cross-sectional study using data of the Korean National Health and Nutrition Examination Survey (KNHANES), conducted by the Korean Centers for Disease Control and Prevention in South Korea. We used data of both the KNHANES V (2010–2012) and KNHANES VI (2013–2015) surveys conducted in South Korea. Of a total 41,102 participants, we included 20,295 participants, aged ≥18 years, for whom results of both urinalysis and serum 25-hydroxyvitamin D (25(OH)D) levels were available. After excluding 55 women who were menstruating at the time of examination, a total 20,240 participants (49.2% of the total population surveyed) were analyzed in the present study.

### Study variables

Demographic variables were collected during health interviews, including age, sex, menopause status, alcohol consumption, and smoking status. Alcohol consumption was defined as drinking once or more per month. Smoking status was classified as nonsmoker, former smoker, or current smoker. Information was also obtained about underlying comorbidities including hypertension, diabetes, and cardiovascular disease. Weight (kg) and height (cm) were measured with participants wearing a gown and no shoes. Body mass index was calculated as weight (kg) divided by square of height (m^2^). Body mass index < 18.5 kg/m^2^, 18.5–22.9 kg/m^2^, 23.0–24.9 kg/m^2^, and ≥ 25.0 kg/m^2^ were defined as underweight, normal weight, overweight, and pre-obese and obesity, respectively [27]. Blood pressure was measured with patients at rest. Participants were defined as having hypertension with systolic blood pressure ≥ 140 mmHg, diastolic blood pressure ≥ 90 mmHg, or a history of taking blood pressure lowering agents. Fasting blood samples were collected during health examination surveys. The samples were refrigerated and transported to the designated central laboratory (NeoDin Medical Institute, Seoul, Korea). Fasting glucose levels were measured using the enzymatic UV (hexokinase) method with a Hitachi 7600 automated analyzer (Hitachi, Tokyo, Japan). Participants with diabetes were defined as those with a fasting glucose level of ≥126 mg/dL or taking diabetes medication or insulin. A fasting glucose level between 100 mg/dL and 125 mg/dL was defined as impaired fasting glucose status. Hypercholesterolemia was defined in participants with total fasting cholesterol level ≥ 240 mg/dL or taking cholesterol lowering agents. Total cholesterol was measured using an enzymatic method and a Hitachi 7600–210 analyzer (Hitachi). Serum hemoglobin levels were measured using the SLS hemoglobin detection method with a XE-2100D analyzer (Sysmex, Tokyo, Japan), and anemia was defined as a hemoglobin level <  13 g/dL for men and < 12 g/dL for women. Serum creatinine levels were measured by the Jaffe rate-blanked and compensated method using the Hitachi 7600–210 analyzer. The estimated glomerular filtration rate was calculated using the Chronic Kidney Disease Epidemiology Collaboration equation [28]. Serum 25(OH)D levels were measured using radioimmunoassay with a 1470 WIZARD gamma counter (PerkinElmer Finland Oy, Finland) with a 25-hydroxyvitamin D 125I RIA kit (DiaSorin Corp., Stillwater Minnesota, USA). We defined serum 25(OH)D inadequacy as serum 25(OH)D level <  30 ng/mL and deficiency as < 20 ng/mL [29]. Random early morning urine samples were collected, whenever possible. All urine samples were refrigerated and transported to the central laboratory (NeoDin Medical Institute). The results of dipstick tests were scored from negative to + 4. Hematuria, proteinuria, and glycosuria were defined as ≥1+ on a dipstick test.

### Statistical analysis

IBM SPSS version 20.0 (IBM Corp., Armonk, NY, USA) was used for all analyses. Continuous variables including age, height, body mass index, blood pressure, fasting blood glucose, serum hemoglobin, and estimated glomerular filtration rate showed normal distributions and were presented as mean value and standard deviation. However, serum 25(OH)D levels showed a non-normal distribution, and were therefore expressed as median value and interquartile range. A logistic regression analysis was used to calculate odds ratios (ORs) and 95% confidence intervals for the risk of hematuria. Multivariate logistic regression was conducted after adjusting all covariates, such as comorbidities and laboratory findings. A nonlinear relationship between 25(OH)D and risk of hematuria was examined using the cubic spline regression model. A *P* value < 0.05 was considered significant.

In this study, subsequent analyses according to sex and menopausal status were conducted to see the risk difference in hematuria. Predicted probability plot of hematuria was drawn according to sex using cubic spine regression model and multivariate logistic regression was conducted according to sex and menopausal status in women.

## Results

### Baseline characteristics

Of a total 20,240 study participants, 10,847 (53.6%) were women and 5388 (26.6%) were identified as postmenopausal. The mean participant age was 49 ± 16.3 years and mean estimated glomerular filtration rate was 88 ± 17.4 mL/min/1.73 m^2^. There were a total 3144 (15.5%) participants with hematuria, and the mean serum 25(OH)D level was 17.4 ± 6.2 ng/mL (median 16.6 ng/mL (13.1–20.8 ng/mL)). Among the total participants, 19,427 (96.0%) had serum 25(OH)D levels < 30 ng/mL, and 14,373 (71.0%) had levels below 20 ng/mL. Blood pressure corresponded to prehypertension in 4845 (23.9%) participants, and 6137 (30.3%) participants had hypertension. A total 3751 (18.5%) patients were diagnosed as having impaired fasting glucose and 1969 (9.7%) patients had diabetes mellitus. A total 503 (2.5%) participants showed glycosuria and 228 (1.1%) proteinuria. Other demographic and laboratory findings are shown in Table 1.

**Table 1:** Baseline characteristics of the study population

	Total (*n* = 20,240)	Normal (≥ 30 ng/mL) (*n* = 813)	Inadequacy (< 30 ng/mL) (*n* = 19,427)	*P*
Age (years)	49.4 ± 16.3	57.9 ± 14.1	49.1 ± 16.3	< 0.001
Female (%)	53.6	46.6	53.9	< 0.001
Post-menopausal women (%)	26.6	26.2	36.9	< 0.001
Body mass index (kg/m^2^)	23.7 ± 3.4	23.4 ± 3.1	23.7 ± 3.4	0. 006
Obesity classification (%)				0.026
Under weight	4.1	3.4	4.1	
Normal weight	39.1	41.4	39.0	
Over weight	24.0	26.7	23.9	
Obesity	32.8	28.4	33.0	
Alcohol (%)	72.8	68.2	73.0	0.003
Smoking (%)				0.010
None	57.9	53.0	58.1	
Former smoker	20.5	24.1	20.3	
Current smoker	21.6	22.9	21.6	
Systolic blood pressure (mmHg)	119.6 ± 17.1	123.3 ± 17.5	119.4 ± 17.1	< 0.001
Diastolic blood pressure (mmHg)	76.2 ± 10.5	76.9 ± 10.3	76.2 ± 10.5	0.066
Blood pressure status (%)				< 0.001
Prehypertension	25	27.3	24.9	
Hypertension	31.7	41.2	31.3	
Fasting blood glucose (mg/dL)	98.1 ± 21.7	99.7 ± 19.6	98.0 ± 21.8	0.016
Impaired glucose tolerance (%)				< 0.001
Impaired fasting glucose	19.7	19.6	22.7	
Diabetes mellitus	10.4	10.2	13.4	
Cardiovascular disease (%)	2.6	3.7	2.5	0.063
Hypercholesterolemia (%)	15.0	17.3	14.9	0.071
Hemoglobin (g/dL)	14.1 ± 1.6	14.1 ± 1.5	14.1 ± 1.6	0.511
Anemia (%)	8.0	9.2	7.9	0.229
Estimated GFR (mL/min/1.73 m^2^)	94.7 ± 17.2	83.7 ± 17.7	88.6 ± 17.3	< 0.001
Estimated GFR under 60 mL/min/1.73 m^2^ (%)	3.7	7.3	3.6	< 0.001
Serum 25(OH)D (ng/mL)^a^	16.6 (13.1–20.8)	33.1 (31.4–35.7)	16.3 (12.9–20.2)	< 0.001
Glycosuria (%)	2.5	2.1	2.5	0.534
Proteinuria (%)	1.1	0.6	1.1	0.215
Hematuria (%)	15.5	12.3	15.7	0.011

*GFR* glomerular filtration rate, *25(OH)D* 25-hydroxyvitamin D

^a^Data are expressed as the median (interquartile range) when the data distribution was skewed

### Factors associated with hematuria

A univariate logistic regression analysis was conducted to examine the association between the covariates and hematuria (Table 2). Age > 30 years, female sex and especially postmenopausal status, hypertension, hypercholesterolemia, anemia, 30–60 mL/min/1.73 m^2^ of estimated glomerular filtration rate, and proteinuria were associated with risk of hematuria. Drinking alcohol, former or current smoker, diabetes mellitus, and glycosuria showed a negative relationship with hematuria. These covariates were adjusted in subsequent multivariate regression analyses.

**Table 2:** Odds ratios for hematuria of baseline variables

Variables	OR (95% CI)	*P*
Age (years)		
< 20	1 (reference)	
20–29	1.14 (0.780–1.651)	0.508
30–39	1.51 (1.054–2.166)	0.025
40–49	2.21 (1.550–3.162)	< 0.001
50–59	2.53 (1.776–3.605)	< 0.001
60–69	2.62 (1.835–3.732)	< 0.001
≥ 70	3.72 (2.604–5.314)	< 0.001
Gender		
Male	1 (reference)	
Female	2.60 (2.390–2.825)	< 0.001
Menopausal status		
Pre-menopause	1 (reference)	
Post-menopause	3.06 (2.782–3.356)	< 0.001
Body mass index (kg/m^2^)		
< 18.5	1 (reference)	
18.5–22.9	1.04 (0.857–1.271)	0.673
23.0–24.9	0.94 (0.769–1.157)	0.573
**≥** 25.0	0.94 (0.771–1.149)	0.551
Alcohol		
(−)	1 (reference)	
(+)	0.68 (0.629–0.742)	< 0.001
Smoking		
None	1 (reference)	
Former smoker	0.53 (0.470–0.587)	< 0.001
Current smoker	0.62 (0.561–0.689)	< 0.001
Hypertension		
None	1 (reference)	
Prehypertension	0.91 (0.910–1.111)	0.913
Hypertension	1.20 (1.096–1.311)	< 0.001
Diabetes mellitus		
None	1 (reference)	
Impaired fasting glucose	1.10 (0.996–1.209)	0.061
Diabetes mellitus	0.67 (0.579–0.777)	< 0.001
Cardiovascular disease		
(−)	1 (reference)	
(+)	1.19 (0.944–1.499)	0.141
Hypercholesterolemia		
(−)	1 (reference)	
(+)	1.13 (1.020–1.261)	0.020
Anemia		
(−)	1 (reference)	
(+)	1.57 (1.384–1.778)	< 0.001
Estimated GFR (mL/min/1.73 m^2^)		
**≥** 60.0	1 (reference)	
30.0–59.9	1.35 (1.117–1.627)	0.002
< 30.0	1.55 (0.739–3.248)	0.247
Glycosuria		
(−) or trace	1 (reference)	
**≥** 1+	0.62 (0.465–0.829)	< 0.001
Proteinuria		
(−) or trace	1 (reference)	
**≥** 1+	5.12 (3.940–6.663)	< 0.001

*OR* odds ratio, *CI* confidence interval, *GFR* glomerular filtration rate

### Association between serum vitamin D level and hematuria

**Fig. 1:**
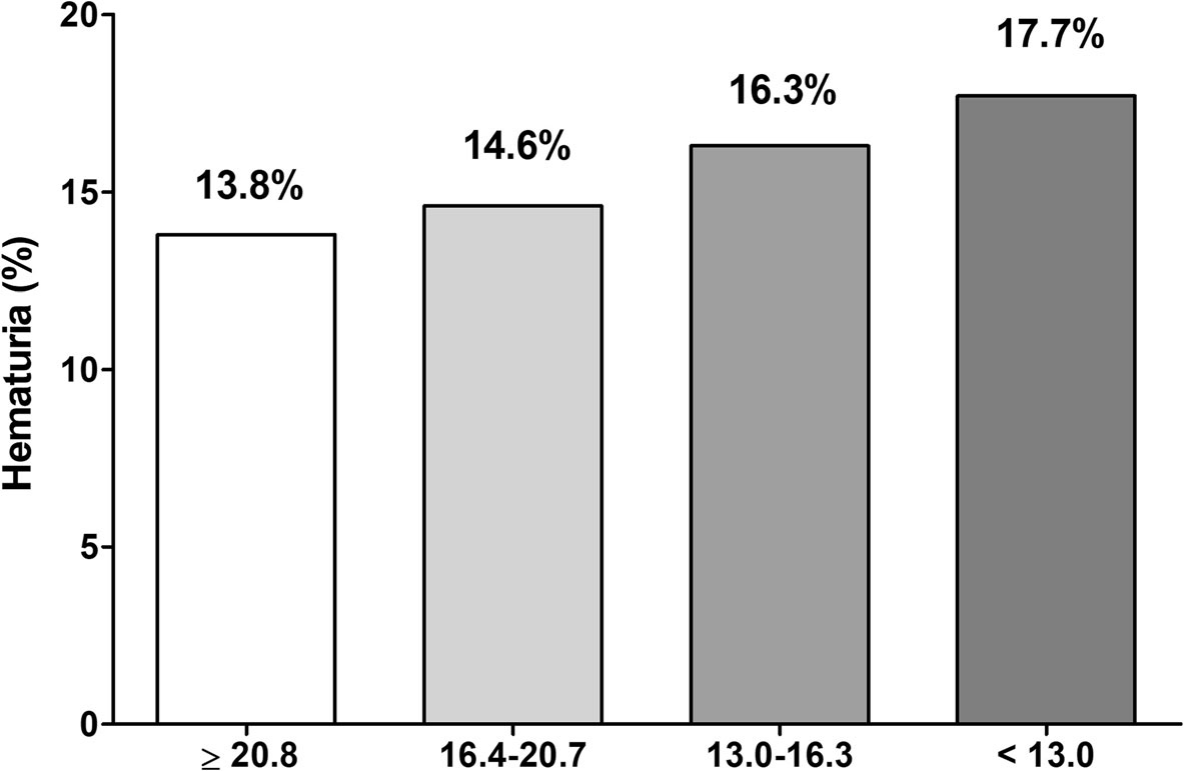
Prevalence of hematuria according to serum 25-hydroxyvitamin D level

As shown in Fig. 1, the prevalence of hematuria increased in proportion to lower 25(OH)D levels. A total 13.8% of participants in the 4th quartile of serum 25(OH)D (≥ 20.8 ng/mL) showed hematuria whereas the prevalence of hematuria increased from the 3rd to 1st quartiles, as follows: 14.6% in the 3rd quartile (16.4–20.7 ng/mL), 16.3% in the 2nd quartile (13.0–16.3 ng/mL), and 17.7% in the 1st quartile (< 13.0 ng/mL) (*P*_trend_ <  0.001). In univariate analysis, the 3rd and 4th quartiles showed a higher risk of hematuria than the 1st quartile: OR 1.20 (1.072–1.336) and OR 1.35 (1.210–1.501) in the 3rd and the 4th quartiles, respectively. When comparing the groups with 25(OH)D levels < 30 ng/mL and ≥ 30 ng/mL, the lower group showed a higher OR of hematuria (1.33 (1.071–1.639)) than the higher group (*P* = 0.010). When vitamin D deficiency was defined as < 20 ng/mL, the deficient group showed a higher OR of hematuria (1.20 (1.102–1.309)) than the higher group (*P* <  0.001). These differences were also significant despite adjusting for multiple covariates which were significant in the univariate analysis (Table 3).

**Table 3:** Odds ratios for hematuria according to serum 25-hydroxyvitamin D levels

Groups	Univariate		Multivariate^a^	
	OR (95% CI)	*P*	OR (95% CI)	*P*
Quartiles				
1st quartile (≥ 20.8 ng/mL)	1 (reference)		1 (reference)	
2nd quartile (16.4–20.7 ng/mL)	1.07 (0.960–1.196)	0.219	1.14 (1.014–1.284)	0.028
3rd quartile (13.0–16.3 ng/mL)	1.20 (1.072–1.336)	0.001	1.26 (1.114–1.415)	< 0.001
4th quartile (< 13.0 ng/mL)	1.35 (1.210–1.501)	< 0.001	1.40 (1.240–1.572)	< 0.001
Vitamin D inadequacy				
Normal (≥ 30 ng/mL)	1 (reference)		1 (reference)	
Inadequacy (< 30 ng/mL)	1.33 (1.071–1.639)	0.010	1.44 (1.150–1.800)	0.001
Vitamin D deficiency				
Normal (≥ 20 ng/mL)	1 (reference)		1 (reference)	
Deficiency (< 20 ng/mL)	1.20 (1.102–1.309)	< 0.001	1.25 (1.137–1.373)	< 0.001

^a^Adjusted for age, gender, alcohol, smoking, diabetes, hypertension, hypercholesterolemia, anemia, chronic kidney disease, glycosuria, and proteinuria

### Subgroup analysis according to sex and menopausal status

**Fig. 2:**
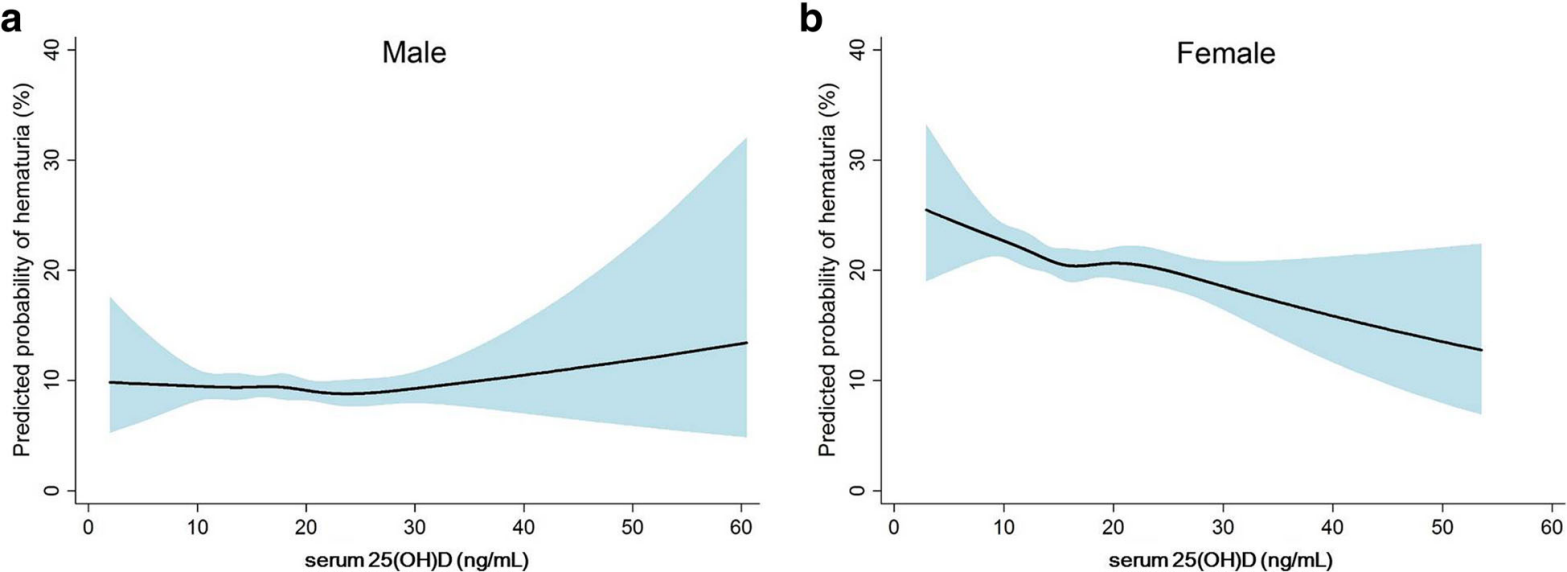
Nonlinear relationship between serum 25-hydroxyvitamin D and risk of hematuria in male (**a**) and female (**b**) subjects

Because the risk of several diseases differs according to sex and menopausal status [10, 25, 30], subsequent analyses were conducted after stratification by these factors. Figure 2 shows the predicted probability plot of hematuria according to sex. The linear relationship seemed to be more dominant in women than in men. When multiple covariates were adjusted, the low 25(OH)D groups (inadequate or deficient) showed higher ORs of hematuria than the high 25(OH)D groups for both sexes. According to menopausal status, no relationship was found among premenopausal women, however, the relationship was significant in postmenopausal women (Table 4).

**Table 4:** Risk of hematuria after stratification by sex and menopausal status

	Male		Female		Pre-menopausal females		Post-menopausal females	
	OR (95% CI)^a^	*P*	OR (95% CI)^a^	*P*	OR (95% CI)^a^	*P*	OR (95% CI)^a^	*P*
Quartiles								
1st quartile	1 (reference)		1 (reference)		1 (reference)		1 (reference)	
2nd quartile	1.23 (1.014–1.498)	0.036	1.09 (0.941–1.266)	0.247	0.94 (0.726–1.225)	0.659	1.18 (0.986–1.418)	0.071
3rd quartile	1.38 (1.121–1.706)	0.002	1.20 (1.035–1.386)	0.015	1.12 (0.875–1.433)	0.369	1.19 (0.991–1.437)	0.062
4th quartile	1.35 (1.080–1.695)	0.007	1.38 (1.200–1.593)	< 0.001	1.15 (0.905–1.460)	0.253	1.58 (1.320–1.892)	< 0.001
Vitamin D inadequacy								
Normal	1 (reference)		1 (reference)		1 (reference)		1 (reference)	
Inadequacy	1.32 (0.915–1.889)	0.139	1.50 (1.127–1.989)	0.005	1.10 (0.597–2.019)	0.764	1.62 (1.177–2.237)	0.003
Vitamin D deficiency								
Normal	1 (reference)		1 (reference)		1 (reference)		1 (reference)	
Deficiency	1.26 (1.077–1.481)	0.004	1.24 (1.101–1.393)	< 0.001	1.15 (0.933–1.419)	0.188	1.29 (1.123–1.491)	< 0.001

*OR* odds ratio, *CI* confidence interval

^a^Adjusted for age, alcohol, smoking, diabetes, hypertension, hypercholesterolemia, anemia, chronic kidney disease, glycosuria, and proteinuria

## Discussion

Vitamin D deficiency and hematuria are important public health problems with high incidence in the general population, and both may be related to more severe diseases. However, there have been no studies conducted to investigate the relationship between vitamin D deficiency and hematuria. We addressed this question in the present study, using data of the KNHANES nationwide population-based survey. The risk of hematuria increased with decreased serum 25(OH)D levels. This increasing risk of hematuria was seen for both vitamin D inadequacy and deficiency, particularly in postmenopausal women. Nevertheless, this relationship was only significant after menopause in women.

Previous studies have reported the correlations between vitamin D deficiency and various diseases wherein hematuria is one of the disease signs [22, 24, 31, 32]. Because vitamin D enhances the absorption of calcium from the intestine and stimulates bone absorption to physiologically increase serum calcium levels [2], it is plausible that vitamin D might increase the risk of urinary stones, thereby leading to hematuria. However, the evidence is insufficient owing to the observational nature of conducted studies [33, 34], and there are contradictory reports in which the risk of calcium-based urinary stones is higher with vitamin D deficiency [22, 35].

Vitamin D has an important role in the immune system via controlling the expression of many immunologic factors. As a result, an association between vitamin D deficiency and risk of urinary tract infection has been reported [23, 36, 37]. One study showed that premenopausal women had a 4-fold increased risk of recurrent urinary tract infection with serum 25(OH)D levels < 15 ng/mL [23], and the correlation between vitamin D deficiency and urinary tract infection has been documented in children and kidney transplant recipients [36, 37]. Because hematuria is one sign of urinary tract infection, the present results may be attributable to the above mechanism.

Vitamin D deficiency is related to the progression of kidney disease via both direct and indirect effects. End-stage renal disease and proteinuria are more prevalent in individuals who are deficient in vitamin D [20, 21, 38]. In a cross-sectional analysis of patients with polycystic kidney disease, kidney volumes were larger in individuals with vitamin D deficiency [31]. Animal studies have showed that low vitamin D levels are correlated with podocyte loss and development of glomerulosclerosis [39]. An acute kidney injury model demonstrated that vitamin D deficiency induces tubulointerstitial damage and fibrosis and diminishes renal vascularity, which finally leads to chronic change [40]. Vitamin D deficiency is additionally linked to activation of the renin–angiotensin system, promoting endothelial damage and the progression of diabetes [41].

Vitamin D deficiency is related to high incidence and aggressiveness of various malignancies [8, 10, 42] that have been documented in the urological system, such as renal cell carcinoma [24] and bladder cancer [32]. Various possible antineoplastic mechanisms of active vitamin D have been suggested. Active vitamin D can regulate transcription of anticancer target genes that induce apoptosis and differentiation and inhibit proliferation, inflammation, angiogenesis, invasion, and metastasis of cancer cells [43]. Vitamin D regulates signaling pathways such as the Wnt/β-catenin, estrogen receptor, and androgen receptor in the colon, breast, and prostate, respectively, which subsequently affect the growth of cancer in each tissue [43]. Additionally, microRNA can mediate the antineoplastic functions of vitamin D [43]. Collectively, the above mechanisms support the present study results.

The subsequent analysis showed that the correlation between hematuria and vitamin D deficiency was predominant in postmenopausal women but not in premenopausal women. The different effects of vitamin D deficiency according to menopausal status have been previously reported [10, 26, 30], but the mechanisms have not been clearly determined. Vitamin D is one of the steroid hormones and it is closely related to sex hormones such as estrogen and testosterone, levels of which may vary with menopausal status, thereby affecting the relationship with disease risk. Further fundamental studies are needed to confirm the different effects of vitamin D on hematuria according to menopausal status.

This study has several limitations. We used one-time spot urine samples and defined presence of hematuria as ≥1+ on a dipstick test. Owing to the possibility of a false positive or false negative on a single test, this approach might have resulted in incorrectly grouped participants. Furthermore, positive dipstick test does not always mean hematuria but may reflect the presence of heme pigment which can be positive in the condition of red blood cell lysis or myositis. Accordingly, using the dipstick test alone may result in false-positivity. Another major limitation is that, we could not obtain information on the cause of hematuria and other laboratory results (e.g., calcium, phosphorous, parathyroid hormone and 1,25 OH Vitamin D level) which may have an interaction in the relationship results. Because the study design was cross-sectional, there is a lack of information about whether the effects of vitamin D deficiency on hematuria eventually lead to occurrence of disease and alter patient prognosis.

Our study is the first to address the correlation between vitamin D deficiency and hematuria risk using a large nationwide cohort. Despite adjusting for several covariates that might affect the presence of hematuria, participants who had inadequate or deficient vitamin D levels had a higher risk of hematuria than participants with normal levels. Further physiological and epidemiological studies are required to find out the underlying mechanisms and whether the supplemental vitamin D would be beneficial in various diseases related to hematuria.

## Conclusions

Vitamin D deficiency and hematuria are both common health problem in general population. The association between vitamin D deficiency and hematuria was noticed in this study, particularly in postmenopausal women. Patients with vitamin D deficiency should be concern about the risk of hematuria and related disease.

## Abbreviations

25(OH)D: Serum 25-hydroxyvitamin D
KNHANES: Korean National Health and Nutrition Examination Survey
OR: Odds ratio
